# Plasmodium malaria and antimalarial antibodies in the first year of life

**DOI:** 10.1017/S0031182015001626

**Published:** 2016-01-08

**Authors:** KATHERINE R. DOBBS, ARLENE E. DENT

**Affiliations:** 1 Division of Pediatric Infectious Diseases, Department of Pediatrics, Rainbow Babies and Children's Hospital, Cleveland, OH, USA; 2 Center for Global Health and Diseases, Case Western Reserve University, Cleveland, OH, USA

**Keywords:** Infant, malaria, antibodies, pregnancy-associated malaria

## Abstract

Malaria is one of the most serious infectious diseases with most of the severe disease caused by *Plasmodium falciparum* (Pf). Naturally acquired immunity develops over time after repeated infections and the development of antimalarial antibodies is thought to play a crucial role. Neonates and young infants are relatively protected from symptomatic malaria through mechanisms that are poorly understood. The prevailing paradigm is that maternal antimalarial antibodies transferred to the fetus in the last trimester of pregnancy protect the infant from early infections. These antimalarial antibodies wane by approximately 6 months of age leaving the infant vulnerable to malaria, however direct evidence supporting this epidemiologically based paradigm is lacking. As infants are the target population for future malaria vaccines, understanding how they begin to develop immunity to malaria and the gaps in their responses is key. This review summarizes the antimalarial antibody responses detected in infants and how they change over time. We focus primarily on Pf antibody responses and will briefly mention *Plasmodium vivax* responses in infants.

## INTRODUCTION

*Plasmodium falciparum* (Pf) malaria is one of the most important paediatric infectious diseases estimated to kill over 600 000 people annually, most of whom are children younger than 5 years of age (WHO, 2014). Yet, newborns and young infants (less than 6 months of age) are thought to be relatively protected from symptomatic malaria (Covell, 1950; Wagner *et al.* 1998; Riley *et al.* 2001; Apinjoh *et al.* 2015). This protection historically has been thought to be primarily mediated by maternal antimalarial IgG antibodies transferred to the fetus in the last trimester of pregnancy. Antibodies play an important role in the host defence against malaria. This was demonstrated when serum transferred from healthy malaria immune adults to hospitalized children with malaria resulted in the rapid amelioration of symptoms and parasitaemia (Cohen *et al.* 1961; McGregor, 1964). Malaria targets of protective antibodies include proteins expressed on the sporozoite needed for hepatocyte invasion, proteins expressed on the merozoite surface important for invasion of erythrocytes and variant surface antigens (VSAs) trafficked to the surface of the infected erythrocyte important for sequestration and pathogenesis (Richards and Beeson, 2009; Dups *et al.* 2014; Smith, 2014). Antimalarial IgG antibodies may function to block sporozoite invasion of hepatocytes and merozoite invasion of erythrocytes, opsonize merozoites and infected erythrocytes expressing VSA on their surface for phagocytosis, and fix and activate complement on the merozoite surface with resultant parasite lysis (Hill *et al.* 2013). An increasing number of Pf antigens have been identified as relevant to naturally acquired immunity and are considered potential vaccine targets (Richards *et al.* 2013; Osier *et al.* 2014; Dent *et al.* 2015), but how and whether antibodies to these antigens are acquired during the first year of life is not well described. With this review, we focus on maternal antibodies directed against Pf targets and their possible role in protecting the infant from malaria, infant susceptibility to malaria as maternal antibodies wane and infant-generated antimalarial antibodies as a result of malaria infection.

## PF MALARIA INFECTION IN NEONATES AND INFANTS

Infants living in malaria endemic areas are relatively protected from clinical malaria during the first 6 months of life. However, there are instances of malaria infection in neonates. For example, congenital malaria occurs after transmission from the mother through the placenta just before or during delivery (though earlier in pregnancy cannot be excluded), with detection of asexual parasites in cord blood or in neonatal peripheral blood within the first week of life (Malhotra *et al.* 2006; Falade *et al.* 2007). Neonatal malaria occurs during the first 28 days of life and is due to an infective mosquito bite after birth. Both congenital and neonatal malaria infections are considered to be very rare (Covell, 1950; Bruce-Chwatt, 1952). Though existing data on prevalence and burden of disease in young infants are still limited and often contradictory (Mwaniki *et al.* 2010), it appears that while subclinical asymptomatic infection is not uncommon, clinical disease due to Pf infection is quite rare in infants under 5 months of age (Sehgal *et al.* 1989; McGuinness *et al.* 1998; Snow *et al.* 1998; Franks *et al.* 2001). Several studies in sub-Saharan Africa demonstrate an increase in malaria prevalence and risk for clinical disease after the ages of 3–6 months (Bruce-Chwatt, 1952; Gottschau and Hogh, 1995; McGuinness *et al.* 1998; Snow *et al.* 1998; Afolabi *et al.* 2001).

In malaria endemic regions where mothers have existing immunity, Pf infection in young infants (less than 6 months) is characterized by low parasitaemia, often <100 parasites *µ*L^−1^ of blood (Sehgal *et al.* 1989; McGuinness *et al.* 1998; Afolabi *et al.* 2001; Falade *et al.* 2007; Mwaniki *et al.* 2010). Such infections are frequently transient, with spontaneous clearance of parasites occurring more quickly than in older infants and children, suggesting that young infants possess some mechanisms for controlling and clearing parasitaemia (Kitua *et al.* 1996; McGuinness *et al.* 1998; Franks *et al.* 2001; Falade *et al.* 2007). Young infants are less likely to be febrile with Pf infection, though the parasite density thresholds for fever appears to be lower in infants (estimated at 100 parasites *µ*L^−1^) than in children older than 12 months (3500 parasites *µ*L^−1^) (McGuinness *et al.* 1998). However, very high parasite densities have been detected in afebrile, asymptomatic infants, suggesting that failure to mount a febrile response to Pf infection may be due to a specific mechanism, such as lack of immunologic priming of T cells needed to produce pro-inflammatory cytokines (McGuinness *et al.* 1998; Riley, 1999). Clinical manifestations of symptomatic malaria infection in neonates and young infants are non-specific and difficult to distinguish from other diseases, such as bacterial sepsis. Signs and symptoms include fever, anaemia, pallor, splenomegaly, lethargy, poor feeding, diarrhoea, respiratory distress, cyanosis, hepatomegaly, jaundice and seizures (Ibhanesebhor, 1995; Afolabi *et al.* 2001; Mwaniki *et al.* 2010).

This relative protection against clinical Pf malaria and progression to severe disease in young infants may be explained by a number of potential immunologic, physiologic and environmental mechanisms. Studies of the biting habits of anopheline mosquitoes show that infants are fed on less frequently than larger adults and older children, which may offer some protection against infection (Muirhead-Thomson, 1951; Port, 1980). Diet may also play an important role in protection. Breast milk constituents such as lactoferrin and secretory IgA have been shown to inhibit parasite growth *in vitro* (Kassim *et al.* 2000). Parasite replication depends on an external source of the nutrient para-aminobenzoic acid, which is low in breast milk (Kicska *et al.* 2003). The presence of fetal haemoglobin (HbF) may provide a physiologic mechanism for protection against clinical malaria. Some studies suggest that parasite growth is restricted in erythrocytes containing higher levels of HbF (Pasvol *et al.* 1977). A transgenic mouse model of adult persistence of HbF demonstrated delayed parasite development and protection from severe disease (Shear *et al.* 1998). In a more recent study, Amaratunga *et al.* showed that Pf parasites invaded and grew normally in cord blood erythrocytes, but that parasitized cord blood erythrocytes had impaired cytoadherence properties; the presence of immune IgG further impaired the ability of these parasitized cells to adhere, suggesting that HbF and maternal IgG act cooperatively to protect young infants (Amaratunga *et al.* 2011).

## MATERNAL ANTIMALARIAL ANTIBODIES IN THE YOUNG INFANT: ROLE OF PROTECTION OR MARKER OF EXPOSURE?

Transplacental transfer of maternal IgG antibodies to the fetus occurs primarily in the third trimester and is mediated by the neonatal Fc receptor (Simister, 2003). After birth, maternal antibodies of all isotypes, but primarily IgA, are transferred to infants in breast milk, though these are not systemically absorbed and act primarily in the gut (Van de Perre, 2003). Studies of birth cohorts in sub-Saharan Africa have reported the waning of maternal antimalarial IgG antibodies by 6–9 months of age, which coincides with the period of time in which the risk for malaria infection and clinical disease in infants begins to increase (Achidi *et al.* 1995; Riley *et al.* 2000; Duah *et al.* 2010; Kangoye *et al.* 2014; Nhabomba *et al.* 2014). It has long been proposed that passive transfer of maternal antimalarial antibodies confers protection against clinical disease in young infants (McGregor *et al.* 1970; Logie *et al.* 1973; McGuinness *et al.* 1998). However, the evidence supporting this assumption is lacking. In Table 1, we summarize the evidence in the literature regarding the question: are maternal antimalarial antibodies that are present at the time of delivery (in maternal and/or cord blood) associated with infant malaria protection? The studies referenced in the table focus on Pf infection.

**Table 1.** Maternal antibodies and association with protection against Pf malaria in infants^a^

Antigen	Association	Study location	References
	***Associated with protection***		
MSP1_19_	Increased duration to first infection (113 *vs* 69 days), detected by microscopy	Kenya	Branch *et al*. (1998)
	Protection against clinical disease in the first 12 months of life	Liberia	Hogh *et al*. (1995)
CIDR1*α*	Increased duration to first infection (6 *vs* 3 months), detected by microscopy	Senegal	Khattab *et al*. (2007)
Crude Pf culture antigen	Seropositivity for anti-Pf IgG2 subclass in cord blood associated with protection against infection in first 6 months of life, detected by microscopy (no association seen with anti-Pf IgG1, IgG3 or IgG4)	Cameroon	Deloron *et al*. (1997)
	***Associated with higher risk***		
CSP, MSP1_19,_ MSP2-FC27, Pf155/RESA and crude schizont antigen	Increased risk of infection in first 5 months of life, detected by PCR	Ghana	Riley *et al*. (2000)
MSP-3	Increased risk of febrile malaria in first 24 months of life	Burkina Faso	Kangoye *et al*. (2014)
CSA–VSA	Decreased time to first parasitaemia and with higher parasite density, detected by microscopy	Cameroon	Cot *et al*. (2003)
	***No association***		
Sporozoites	No association with time to first infection, detected by microscopy	Tanzania	Mutabingwa *et al*. (1993)
CSP, LSA1, MSP2	No association with time to first infection, detected by microscopy	Kenya	Zhou *et al*. (2002)
AMA1, MSP2-IC1	No association with risk of infection in first 5 months of life, detected by PCR	Ghana	Riley *et al*. (2000)
CSP, Pf155/RESA	No association with age of onset of clinical malaria	Nigeria	Achidi *et al*. (1996)
Pf155/RESA	No association with risk of infection in first 6 months of life, detected by microscopy	Cameroon	Deloron *et al*. (1997)
GLURP	No association with risk of febrile malaria in first 24 months of life	Burkina Faso	Kangoye *et al*. (2014)
Crude schizont antigen	No association with risk of clinical disease in first 12 months of life in multivariate analysis	Cameroon	Apinjoh *et al*. (2015)

a Maternal antibodies are defined as antimalarial antibodies present at the time of birth in either maternal or cord blood. CSP, circumsporozoite protein; MSP1_19_, merozoite surface protein-1 GPI-anchored 19-kD fragment; CIDR1*α*, cysteine-rich interdomain region of *P. falciparum* 732*var* gene; Pf155/RESA, ring-infected erythrocyte surface antigen; MSP2-FC27, merozoite surface protein-2 from FC27 *P. falciparum* isolate; MSP3, merozoite surface protein-3; CSA–VSA, chondroitin sulphate-A variant surface antigen; LSA1, liver stage antigen-1; AMA1, apical membrane antigen-1; MSP2-IC1, merozoite surface protein-2 from IC1 *P. falciparum* isolate; GLURP, glutamate-rich protein.

Longitudinal studies designed to answer this question vary in regards to antigens studied, reported outcomes (infection *vs* clinical disease), method for detecting parasitaemia, location and transmission intensity. A few of these studies have demonstrated an association between maternal antimalarial antibodies and protection from infection (Deloron *et al.* 1997; Khattab *et al.* 2007), clinical disease (Hogh *et al.* 1995) or both (Branch *et al.* 1998) in infants. However, several others have found an association between maternal antibodies and an increased risk for infection in infants (Riley *et al.* 2000; Cot *et al.* 2003; Kangoye *et al.* 2014), or no association at all (Mutabingwa *et al.* 1993; Achidi *et al.* 1996; Deloron *et al.* 1997; Riley *et al.* 2000; Zhou *et al.* 2002; Kangoye *et al.* 2014; Apinjoh *et al.* 2015). For example, the presence of maternal antibodies against merozoite invasion protein 1 (MSP1_19_) were associated with delayed onset of first infection among a cohort in western Kenya and with protection of infants against clinical disease in Liberia (Gottschau and Hogh, 1995; Branch *et al.* 1998). In a comprehensive study of the role of passively acquired antimalarial antibodies in infants living in Southern Ghana, Riley *et al.* found that antibodies against MSP1_19_ (as well as circumsporozoite surface protein (CSP), MSP2, ring-infected erythrocyte surface antigen (Pf155/RESA) and crude schizont antigen) were positively associated with infection (Riley *et al.* 2000). Other studies in West Africa also found a positive association between maternal antibodies against MSP3 and chondroitin sulphate A (CSA)–VSA and an increased risk for malaria (Cot *et al.* 2003; Kangoye *et al.* 2014). We and these authors conclude that the presence of antimalarial antibodies at birth is a biomarker for intensity of exposure to malaria in infants (Franks *et al.* 2001).

It is important to consider that different antigens have been used as markers of Pf infection so there is very little consistency between studies. In a study of malaria-exposed pregnant women in Senegal, Khattab *et al.* showed that maternal antibodies against the 732var Pf erythrocyte membrane protein 1 (PfEMP1) domain cysteine-rich interdomain region 1*α* (CIDR1*α*) conferred protection against malaria in infants during the first 6 months of life (Khattab *et al.* 2007). In another study of anti-VSA antibodies, Cot *et al.* showed that the presence of maternal antibodies against CSA–VSA was associated with a decreased time to first parasitaemia (Cot *et al.* 2003). These discrepant results are likely explained by the different function of the antigens. CIDR1*α* mediates parasite sequestration by binding to endothelial cell surface receptors, and this domain is expressed in parasite strains relevant to infection and disease in young children (Avril *et al.* 2013; Turner *et al.* 2013; Smith, 2014). Antibodies directed against CSA–VSA are important in response to placental malaria, and thus likely represent recent exposure in mothers of these infants.

Several factors contribute to an infant's risk for malaria, and it is difficult to disentangle any one factor from the others. The waning of maternal antibodies occurs simultaneously with the waning of HbF, changes in infants’ diets, and decreased breastfeeding, all of which might alter risk for malaria (Colombo *et al.* 1976). Transmission intensity and season of birth have been shown to be very important factors in risk for clinical disease (Riley *et al.* 2000; Apinjoh *et al.* 2015). Maternal HIV and placental malaria have also been implicated (Briand *et al.* 2009).

## *PLASMODIUM VIVAX* (PV) INFECTION IN THE NEONATE AND INFANT

Cases of congenital Pv malaria have been described in infants born to mothers without pre-existing antimalarial immunity, mostly in case reports of infants born in non-endemic countries to mothers with a travel history to endemic countries (Del Punta *et al.* 2010). These infants often present several days to weeks after birth with fever, irritability and hepatosplenomegaly, and if untreated, the infection may be rapidly fatal (Menendez and Mayor, 2007). Very little is known about the development of humoral immunity to Pv in the first year of life. The majority of Pv infections occur in Southeast Asia and the Western Pacific, with a significant number of cases also occurring in the Eastern Mediterranean, South and Central America, and Africa (Price *et al.* 2007). Studies performed in Papua New Guinea, Indonesia and Vanuatu demonstrate that in areas endemic for both Pv and Pf, Pv serves as the predominant cause of malaria infection during the first 2 years of life (Maitland *et al.* 1996; Karyana *et al.* 2008; Poespoprodjo *et al.* 2009; Senn *et al.* 2012). In contrast to Pf infection in young infants, which has a low risk for progression to symptomatic or severe disease, Pv infection in infants younger than 3 months of age is a significant cause of morbidity in endemic areas, characterized by higher parasite densities and substantial risk for development of severe disease (Poespoprodjo *et al.* 2009). Limited data exist regarding risk factors for Pv malaria in infants, and as with Pf, it is not known if transfer of maternal antimalarial antibodies confers any protection against Pv infection in infants (Campbell *et al.* 1980). Clinical immunity to Pv appears to develop more rapidly than to Pf, with most children in endemic areas acquiring immunity to Pv by 5 years of age while remaining at risk for Pf illness (Michon *et al.* 2007). Further studies are needed to evaluate how protective immunity develops in infants and to determine which factors might be unique to Pv compared with Pf infant infections.

## PREGNANCY-ASSOCIATED PF MALARIA AND INFANT IMMUNITY

Pregnancy-associated malaria (either peripheral or placental detection of parasites) can have devastating effects on both mother and fetus, including anaemia, stillbirth, low birthweight, intrauterine growth retardation and pre-term delivery (Brabin, 1991; Menendez, 1995; Brabin *et al.* 2004; Nosten *et al.* 2004; Desai *et al.* 2007; Rogerson *et al.* 2007). Some studies have found that infants born to mothers with Pf placental malaria have increased susceptibility to malaria with earlier infections compared with those born to mothers with no evidence of pregnancy-associated malaria (Le Hesran *et al.* 1997; Mutabingwa *et al.* 2005; Schwarz *et al.* 2008; Malhotra *et al.* 2009; Bardaji *et al.* 2011; Le Port *et al.* 2011). The reasons for this are likely multifactorial with maternal parity, receipt of intermittent preventive treatment during pregnancy, use of insecticide treated bed nets and malaria transmission intensity playing important roles (Apinjoh *et al.* 2015).

*In utero* exposure to parasite antigens may induce fetal immune tolerance (Le Hesran *et al.* 1997). In placental malaria, infected erythrocytes sequester in the intervillous blood of the placenta such that the fetus may be exposed to infected erythrocytes or soluble malaria antigens that cross the placenta. May *et al.* (2009) showed that immune-complexed MSP1 transferred from maternal to fetal circulation using an *ex vivo* human placental cotyledon model. Numerous studies have shown that fetal cord blood lymphocytes can have recall responses to specific malaria antigens (Fievet *et al.* 1996; King *et al.* 2002; Malhotra *et al.* 2005, 2009, 2015; Dent *et al.* 2006; Metenou *et al.* 2007) and cord blood can have fetal malaria-specific IgM and IgE, immunoglobulins that do not cross the placenta (Rasheed *et al.* 1995; Xi *et al.* 2003; Apinjoh *et al*. 2015). The consequence of fetal tolerance to malaria antigens is poorly understood, but we have shown that infants exposed to malaria *in utero* but lacking malaria-specific cord blood lymphocyte recall responses (putatively tolerized) make less functional MSP 1_19_ invasion inhibitory antibodies (Dent *et al.* 2006). Bonner *et al*. showed that infants born to mothers with placental malaria had lower antibody responses to multiple malaria antigens, including CSP, EBA175 and MSP2 compared with infants born to placental malaria negative mothers. This difference was apparent especially after 4 months of age when maternal antibodies wane (Bonner *et al.* 2005). Thus, *in utero* exposure to malaria may have detrimental effects on infant antimalarial antibody generation through poorly understood mechanisms. It is likely, this is a generalizable immune consequence as infants born to mothers with placental malaria have increased all-cause mortality (Verhoeff *et al.* 2004; Bardaji *et al.* 2011; Rachas *et al.* 2012). Additionally, malaria putatively tolerized Kenyan infants, those with *in utero* exposure to malaria but lacking cord blood lymphocyte recall responses to malaria antigens, had lower levels of antibodies to diphtheria toxin vaccine compared with those infants who were not exposed to malaria *in utero* (Malhotra *et al.* 2015). Thus, fetal exposure to malaria *in utero* may affect subsequent infant immune responses to malaria and other pathogens.

## ACQUISITION OF ANTIMALARIAL ANTIBODIES IN THE INFANT AFTER 6 MONTHS OF AGE

After maternal antibodies wane, infants exposed to malaria gradually acquire antimalarial antibodies. Evaluation of infant antibody responses to Pf has relied primarily on serologic assays with Pf schizont extract, single recombinant proteins and/or protein domains. Numerous antigens on the surface and within merozoites have been identified as important targets of naturally acquired immunity. Targets include MSPs thought to be important in initial attachment of the merozoite to the erythrocyte, apical membrane antigen (AMA1) implicated in apical reorientation of merozoite, and erythrocyte-binding proteins (e.g. EBA175, EBA140 and EBA181) and reticulocyte-binding homologues (e.g. Rh4 and Rh5) thought to be important in erythrocyte invasion (Cowman and Crabb, 2006). Pre-erythrocytic antigens such as CSP are also important targets as infant trials with the vaccine containing CSP named ‘RTS,S’ have indicated (Rts, 2015). Studies of antimalarial antibodies and protection from malaria in children vary greatly in design, outcome measured (i.e. infection or symptomatic disease), age of children included, transmission intensity and duration of follow-up. Alleles and recombinant protein preparation used also vary widely. A challenge to this review is the paucity of studies conducted in infants. Thus, we extended our review of the literature to include studies that included 12-month-old infants and young children. In multiple studies including infants and young children, serologically measured antibodies against targets such as MSP1, MSP2, MSP3, AMA1, glutamate-rich protein (GLURP) and EBA175 have been associated with protection from clinical malaria (Roussilhon *et al.* 2007; Osier *et al.* 2008; Fowkes *et al.* 2010; Richards *et al.* 2010, 2013). With recent advances in genomics, proteomics and methods for protein expression, an increasing number of putative targets of naturally acquired immunity are being identified. These include hypothetical proteins and targets with no identified function. Osier *et al*. examined antibodies to 36 different proteins in a cohort of Kenyan children who were followed for malaria for 6 months. They found that the breadth and magnitude of antibody responses increased with age, and that the breadth of responses was a better correlate of protection than individual antibody responses (Osier *et al.* 2008). In our microarray study examining antibody responses in a treatment-time to infection study in Kenya, we also found that 1–4-year-old children had the narrowest breadth and the lowest magnitude of antibody responses compared with older children and adults (Dent *et al.* 2015). In our longitudinal infant study in Kenya, 12-month-old infants who had detectable serologically measured antimalarial antibodies had an increased risk of malaria in the subsequent year of life compared with 12-month-old infants with no detectable antimalarial antibodies (Dent submitted). Recently, Stanisic *et al.* (2015) examined antimalarial antibodies in cohorts of 1–4 and 5–14-year-old Papua New Guinean children. They found that young children who had higher levels of antibodies to MSP2, AMA1, EBA175, EBA140 and EBA181 had an increased risk of malaria compared with young children with low or no detectable antimalarial antibodies. Additionally, young children had antimalarial antibodies that were of significantly lower magnitude than those found in protected older (5–14-year-old) children. Results of this study indicate that one of the reasons why antibody responses in young infants represent biomarkers of malaria exposure rather than protection from malaria is related to failure of antibody responses to reach a critical ‘protective’ level, as determined by serology, until age 4 years or older. This is consistent with other studies that have found the magnitude of antibody responses influences protection from malaria (John *et al.* 2005; Osier *et al.* 2008; Courtin *et al.* 2009; Murungi *et al.* 2013). Serologically measured antimalarial antibodies may be a biomarker of exposure rather than protection especially in infants. Serology detects the presence of antibodies recognizing an antigen, but does not measure the function of these antibodies. Despite this, serology is useful to identify and prioritize antigen targets, especially in older protected children and adults, which may be valuable in a multicomponent vaccine, essential for eradicating malaria. Follow-up functional assays are invaluable tools in validating serologically identified targets.

VSAs are considered a major target of naturally acquired immunity (Bull *et al.* 1998; Hviid, 2010; Chan *et al.* 2014) and include PfEMP1, repetitive interspersed family, subtelomeric variable open reading frame and surface-associated interspersed gene family proteins with PfEMP1 being the key target of humoral immunity (Chan *et al.* 2012). PfEMP1, encoded by the *var* multigene family, is highly polymorphic and expression is clonally variant (Smith *et al.* 1995). Antibodies that bind to VSA may function to prevent tissue sequestration by blocking VSA binding to endothelial receptors and facilitating opsonic phagocytosis by monocytes and macrophages. Serology has been used to measure antibody responses to various proteins and protein domains, but the VSA assay utilizes the whole Pf parasite to identify IgG antibodies that recognize the trafficked proteins on the surface of the infected erythrocyte. VSA antibodies are known to be highly parasite isolate-specific among children (Marsh and Howard, 1986; Reeder *et al.* 1994), and may be short-lived (Kinyanjui *et al.* 2003). It is thought that young children develop VSA antibodies to parasites expressing VSA associated with severe disease earlier in childhood than VSA associated with mild or moderate disease (Hviid and Staalsoe, 2004). Studies in infants are limited but Vestergaard *et al*. (2008) showed that infants residing in a low malaria transmission region of Tanzania had low prevalence and magnitude of VSA antibodies compared with infants residing in regions of high transmission. In high transmission areas, VSA antibodies increased with age between 5 and 24 months. Nhabomba *et al.* (2014) found a lack of VSA antibody acquisition in infants up to 2 years of age in Mozambique, though their group previously found that infants acquire VSA antibodies in a study conducted in the same region at an earlier time when malaria transmission was presumably higher (Quelhas *et al.* 2011). A caveat to these observations is that the parasite isolates used in these studies may not accurately represent the circulating parasites at the time the studies were conducted. A study in Papua New Guinea using a PfEMP1 microarray demonstrated that young children (<2 years old) had low prevalence, magnitude and breadth of antibodies compared with older children (Barry *et al.* 2011). In general, VSA antibodies are low in infants and may not be readily acquired in the first year of life depending in large part on the geographical malaria transmission levels.

A better way to evaluate infant immunity may be to use assays that assess the function of the antimalarial antibodies. Antibodies may function by binding to merozoite surface proteins to inhibit erythrocyte invasion and intraerythrocytic growth. These can be measured with the Growth Inhibition Assay (GIA) which quantifies antibody-mediated activity against parasites *in vitro* and has been used to assess vaccine efficacy in animal models and malaria-naïve human volunteers (Singh *et al.* 2006; Dutta *et al.* 2009; Spring *et al.* 2009; Remarque *et al.* 2012; Otsyula *et al.* 2013). In malaria endemic populations, naturally acquired immunity measured by GIA has been associated with protection from infection in children in some studies, but this has not been a consistent finding (Dent *et al.* 2008; McCallum *et al.* 2008; Courtin *et al.* 2009; Crompton *et al.* 2010). Growth inhibitory activity appears to decrease or remain relatively stable with age (Dent *et al.* 2008; McCallum *et al.* 2008; Courtin *et al.* 2009). Few studies have examined GIA in infants specifically. Wilson *et al*. examined GIA in infants from delivery to 12 months of age and found that GIA antibodies decline over time becoming essentially undetectable by 12 months of age (Wilson *et al.* 2013). In contrast, Quelhas *et al*. found detectable GIA in 12-month-old infants that increased by 24 months of age but was not associated with protection from malaria (Quelhas *et al.* 2011). In a longitudinal Kenyan infant study utilizing the MSP1_19_ mediated invasion inhibition assay (MSP1_19_ IIA) which uses transgenic parasites, by 12 months of age, infants had detectable MSP1_19_ IIA that gradually increased by 24–30 months after birth (Dent *et al.* 2006). The MSP1_19_ IIA measures one pathway of invasion inhibition which is only one component of the overall GIA. A drawback of GIA is that the targets of the antibodies that inhibit growth *in vitro* are not known and as with the VSA assay, the parasite isolates used in the GIA may not accurately represent the circulating parasites. Young children may have GIA antibodies, but this may be a reflection of malaria transmission (McCallum *et al.* 2008). Further studies of the acquisition of functional antibodies in young children are needed.

In summary, infants acquire antimalarial antibodies with serologically measurable antibodies possibly acquired earlier than VSA or GIA measured antibodies which may reflect the late and complex acquisition of functional antibodies. Serologically measured antibodies may be a biomarker of exposure and may not protect the young child until a certain magnitude threshold is reached. These observations coupled with the immaturity of the infant immune system until 2 years of age (Jaspan *et al.* 2006) makes understanding how infants and young children acquire immunity a challenge.

### Concluding remarks and future directions

Early infancy is a critical time in the development of immunity to malaria in children born in malaria endemic areas. Antibodies are known to play an important role in host defence against malaria, but the acquisition of antimalarial humoral immunity in infants is not yet well understood. Passively transferred maternal antibodies thought to protect infants younger than 6 months from clinical disease may actually be a biomarker of exposure and risk of infection rather than a correlate of protection. Infants with maternal antimalarial antibodies still become infected with low-grade parasite densities. As maternal antibodies wane, older infants gradually acquire antimalarial antibodies, with increasing breadth and magnitude of responses with age and exposure. Fetal exposure to malaria *in utero* may have an important effect on infant immune responses to malaria and other pathogens. In infants and young children, antibodies detected by serology may be a biomarker of exposure, with protection afforded only after a critical threshold is reached. As infants are a target population for future malaria vaccines, understanding the complexity of achieving protection in the context of an immature immune system must be considered. Further studies and development of novel assays are needed to analyse the functional capabilities of these antibodies and to evaluate the maternal, fetal and neonatal factors leading to the development of protective immunity in infants.
